# Interpreting Radiographs with Concurrently Obtained Patient Photographs

**DOI:** 10.1148/rg.2019180198

**Published:** 2019

**Authors:** Srini Tridandapani, Pamela Bhatti, Richard K. J. Brown, Elizabeth A. Krupinski, Nabile M. Safdar, Eliot L. Siegel, Carson A. Wick

**Affiliations:** Department of Radiology, University of Alabama School of Medicine, 619 19th Street South, JT N455E, Birmingham, AL 35249-6830 (S.T.); Department of Radiology and Imaging Science, Emory University School of Medicine, Atlanta, Ga (S.T., E.A.K., N.M.S.); School of Electrical and Computer Engineering, Georgia Institute of Technology, Atlanta, Ga (S.T., P.B.); Camerad Technologies, LLC, Decatur, Ga (S.T., P.B., C.A.W.); Department of Radiology, University of Michigan, Ann Arbor, Mich (R.K.J.B.); and Department of Diagnostic Radiology, University of Maryland, Baltimore, Md (E.S.). Received July 29, 2018; revision requested September 13 and received September 21; accepted October 29. For this journal-based SA-CME activity, the authors S.T., P.B., and C.A.W. have provided disclosures (see end of article); all other authors, the editor, and the reviewers have disclosed no relevant relationships.

## Abstract

A technology for automatically obtaining patient photographs along with portable radiographs was implemented clinically at a large academic hospital. This article highlights several cases in which image-related clinical context, provided by the patient photographs, provided quality control information regarding patient identification, laterality, or position and assisted the radiologist with the interpretation. The information in the photographs can easily minimize unnecessary calls to the patient’s nursing staff for clarifications and can lead to new methods of physically assessing patients.

## Introduction

Motivated by a need to increase the detection rate for wrong-patient errors in radiology (1–4), we developed a technology to automatically obtain point-of-care patient photographs along with portable radiographs (5–7). Observer studies in controlled laboratory-like settings demonstrated that the addition of patient photographs to portable chest radiographs can increase the detection of wrong-patient errors, without substantially increasing interpretation time (8–11).

We recently implemented this technology in a clinical setting in a live picture archiving and communication system (PACS) at Emory University Hospital in Atlanta, Georgia, and our findings and impressions are described here. The general consent form for treatment at our institution explicitly notes that patient photographs may be obtained either for patient identification or clinical purposes. Therefore, it was deemed by Emory University Hospital’s management group that additional consent to obtain these photographs was not necessary. It should be noted that the photographs are stored within the PACS, which is a Health Insurance Portability and Accountability Act (HIPAA)–compliant environment. It should also be noted that HIPAA considers a patient facial photograph to be an identifier akin to patient name, date of birth, or medical record number.

We describe several cases in which the addition of such point-of-care photographs provided relevant clinical context during interpretation. Implementing such practices can *(a)* provide quality control information regarding patient identification, laterality, and position; *(b)* assist with and add confidence to the interpretation; *(c)* minimize unnecessary calls to the patient’s physicians or nursing staff for clarification, leading to increased interpretation efficiency; and *(d)* lead to new ways of physically assessing patients.

## Camera Technology in Brief

A general overview of the camera technology is provided by Ramamurthy et al (5), and further details regarding its operation have been described by Ramamurthy et al (6) and Tridandapani et al (7). A small camera is mounted on the x-ray head of the portable radiography machine. Each time the x-ray trigger is depressed, the camera is also triggered, enabling the simultaneous acquisition of the photograph and radiograph. This is enabled by a custom-designed switch placed on top of the x-ray trigger. If multiple views are obtained for an examination, then a photograph is obtained for each view.

An off-the-shelf camera with a wide-angle lens (SainSmart Wide Angle; SainSmart, Lenexa, Kans) is used to ensure that the patient’s face is almost always captured when obtaining chest and abdominal radiographs. The camera technology functions seamlessly in the background and does not require any technologist intervention (ie, the technologist does not have to become a photographer).

The camera and camera controller are smart devices that securely communicate through the hospital’s Wi-Fi (or *wi*reless *fi*delity) through a dedicated system integration server (IS). The camera and a Raspberry Pi Zero (Raspberry Foundation, Cambridge, UK) controller are powered by the universal serial bus (USB) port available on the portable radiography machine.

**Figure 1.**
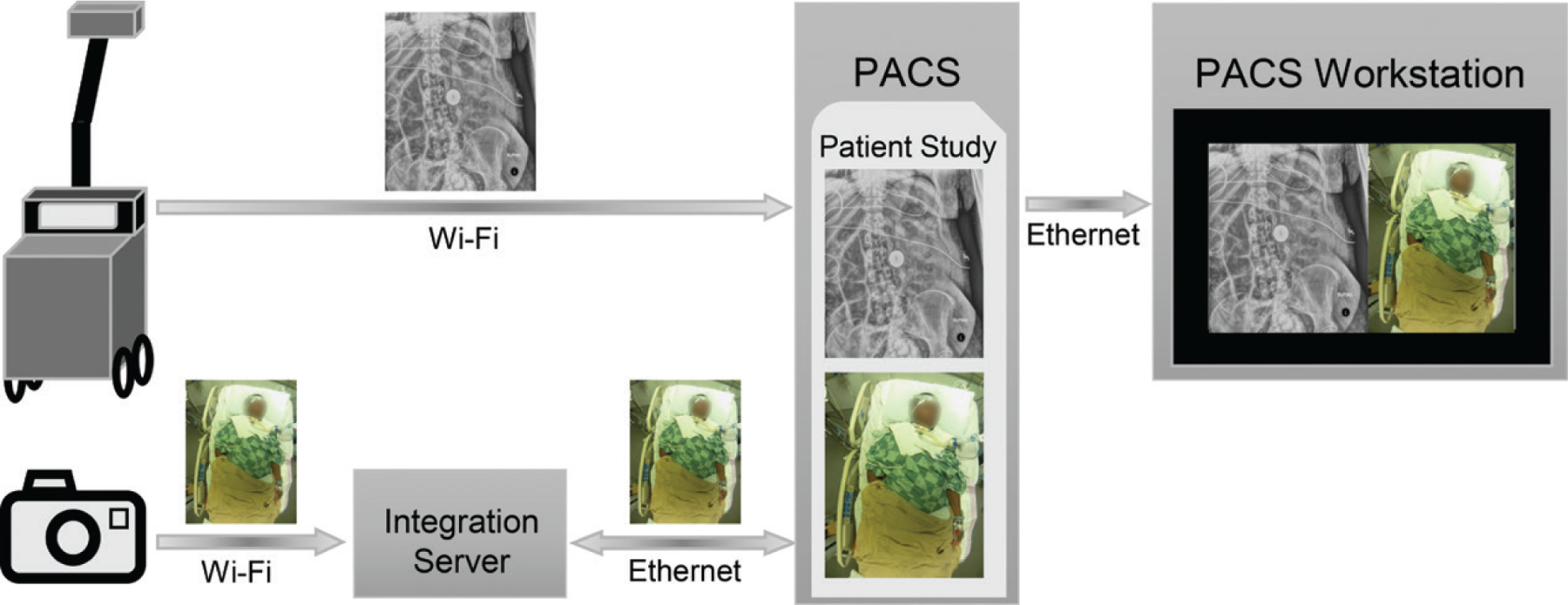
Flow chart shows the system architecture and information flow for simultaneously obtaining photographs and portable radiographs. A portable radiography machine transmits portable radiographs through Wi-Fi to a PACS. The simultaneously acquired photograph is transmitted through Wi-Fi by the smart camera to an IS, along with a time stamp and a machine identifier, which is shared by the portable radiography machine and the camera. The IS queries the PACS through the Ethernet for radiographic studies that correspond to the time stamp and machine identifier. Once it retrieves the Digital Imaging and Communications in Medicine (DICOM) header for the appropriate study, it converts the bitmap image(s) from the camera to a DICOM image using the matching DICOM header as a template, transmitting it to the PACS as a separate series in the same study. The study can be displayed on any PACS workstation, with appropriate custom hanging protocols.

The system architecture and information flow are briefly described in Figure 1. The radiographs are transmitted to the PACS using Wi-Fi without any modification. The photographs are sent through Wi-Fi to the IS with a time stamp and a machine identifier. Both of these attributes are also present within the DICOM header of the radiograph. The IS queries the PACS for radiographic studies that correspond to the specific time stamp and machine identifier. If such a study is present in the PACS, the corresponding DICOM header is retrieved by the IS. The IS then converts the bitmap image(s) into a DICOM format, using the matching DICOM header as a template, and sends them to the PACS as a separate series in the appropriate study. For the matching to work, the clock on each camera is synchronized with the clock on the corresponding portable radiography machine. This can be achieved by having all cameras and portable radiography machines synchronized by a common network–time protocol server.

The study can be viewed on any PACS workstation. Figure 1 shows a possible hanging protocol in which the radiograph is shown on the left side of the display screen and the corresponding photograph is shown on the right side. For our pilot implementation, we did not want to substantially alter the radiologist’s workflow and mandate the viewing of the photographs. Therefore, the photographs were not automatically displayed as soon as the study was opened by the radiologist. Instead, the photographs were added as a separate series at the end of the radiographs, and radiologists accessed the photographs by scrolling through the images.

Reviewing the patient photograph is an optional component of the interpretation, and the information obtained is not recorded in the dictated report. Our goal is to eventually develop, in consultation with radiologists, consensus hanging protocols to accommodate the photographs. This will involve optimization of the initial display size of the photographs so that they provide appropriate information without unnecessarily distracting radiologists from the radiographs. Presently, the display size of the photographs is the same as that of the radiographs.

## Impact of Point-of-Care Photographs on Portable Radiography

In this section, we provide cases in which point-of-care photographs obtained along with portable radiographs were beneficial. To protect patient identity, the patients’ faces and wristbands have been obscured. However, the interpreting physicians were able to see the entire unedited photographs, as they had doctor-patient relationships with the patients.

## Impact on Quality Control

Despite use of the Joint Commission–mandated dual-identifier technique for positive patient identification, wrong-patient errors continue to occur in radiology. The detection of wrong-patient errors was the original motivation for developing this technology. In a previously published retrospective study, the lower bound of the number of wrong-patient errors in radiology was 67 of 1.7 million examinations over a 3.5-year period (an error rate of at least 0.000039) at a large academic institution (2). The Pennsylvania Patient Safety Authority documented that in one year (2009) there were 196 wrong-patient errors in the field of radiology in the state that resulted in serious harm to patients (1).

**Figure 2.**
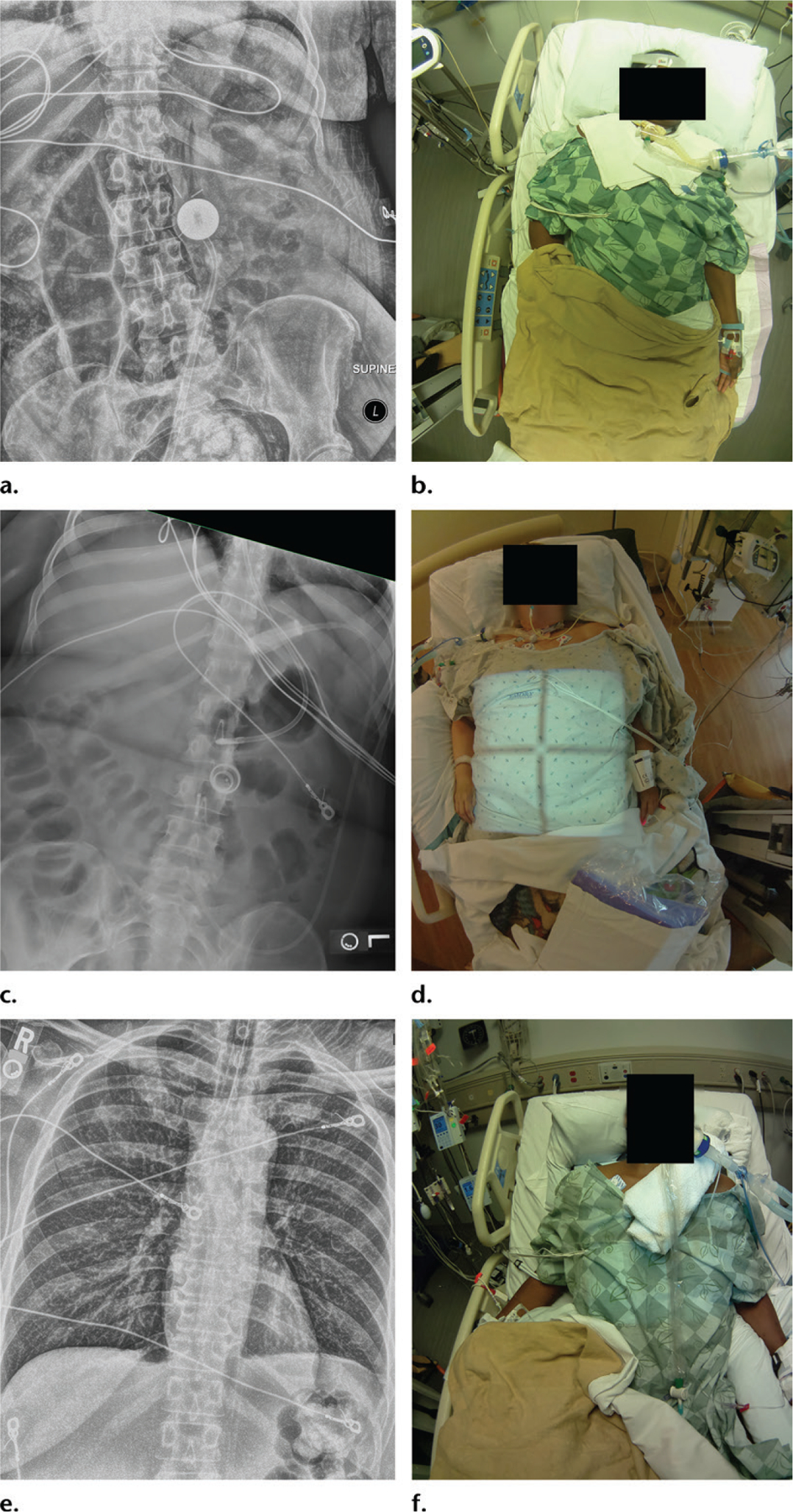
Wrong-patient error. **(a, b)** Portable abdominal radiograph **(a)** and photograph **(b)** were obtained in an African American woman with nausea and vomiting. **(c, d)** Portable radiograph **(c)** and photograph **(d)** obtained 6 days earlier and used as a comparison study show a different patient, a white woman with a gastric band. The differences between the photographs were noted by the interpreting radiology resident. **(e, f)** Portable radiograph **(e)** and photograph **(f)** from a more remote study obtained 8 days earlier show that the patient in **c** and **d** was the wrong patient. This error had not been noticed during the interpretation of **c**, as the interpreting radiologist had not looked at the corresponding photograph **(d)**. The challenge was to determine the identity of the patient depicted in **c** and **d**. The technologist recognized the floor in the room where the study was performed and noticed some features in the background in **d**. The technologist took the photograph **(d)** to the floor, where a nurse was able to identify the patient.

Within the first 350 portable radiographs obtained after this technology was introduced at our institution, we noticed one wrong-patient error. A second error was observed shortly before 8000 such examinations were performed (Fig 2). The system has not been operational long enough to show if there is a statistically significant increase in the detection rate of wrong-patient errors. However, in three prior observer studies performed under various conditions, we demonstrated that the detection rate for simulated wrong-patient errors increased from 12.5% to 64% (8), from 0% to 94.4% (9), and from 31% to 77% (10). Thus, patient photographs obtained at point-of-care portable radiography can help identify wrong-patient errors.

**Figure 3.**
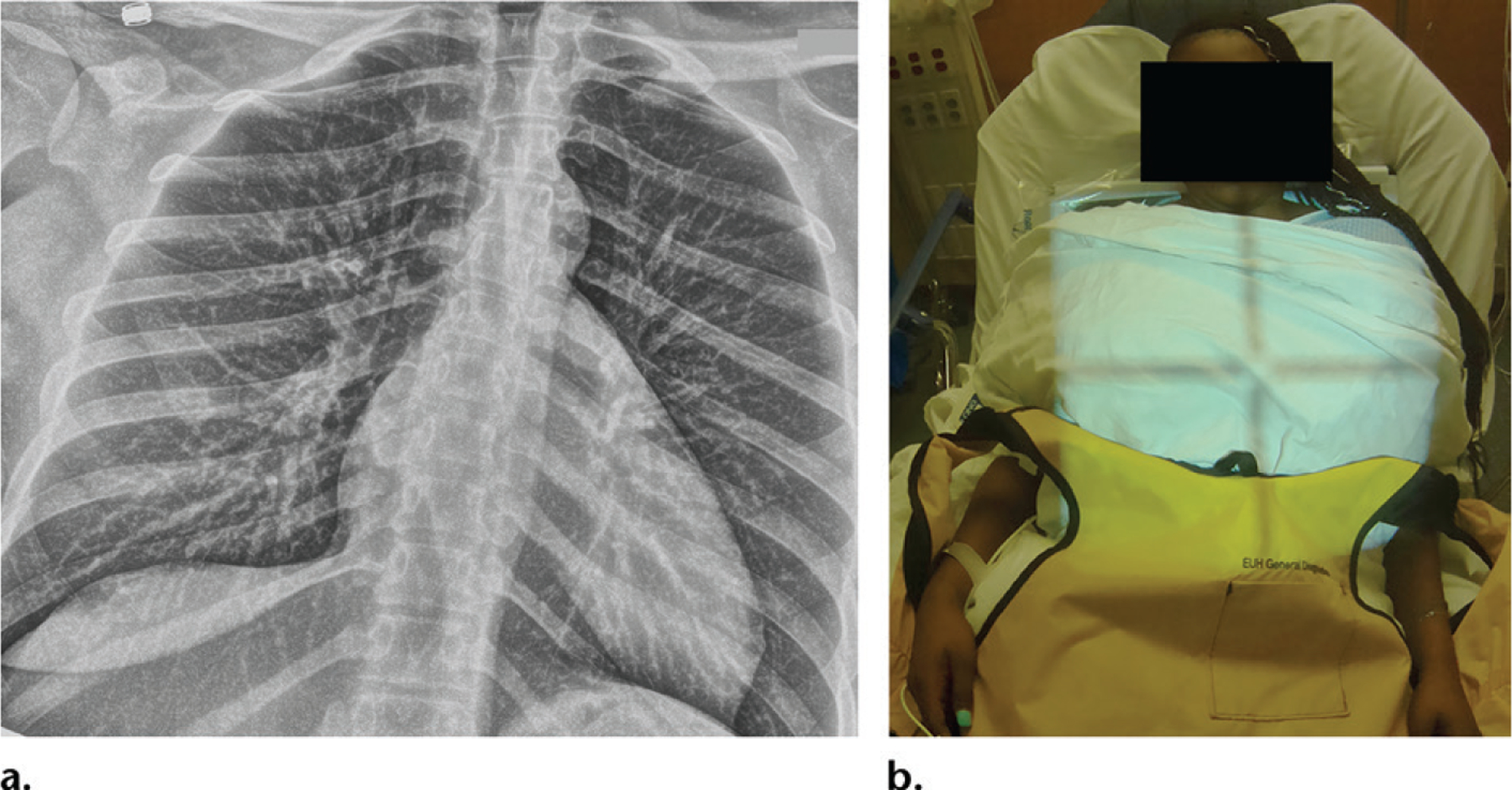
**(a)** Portable chest radiograph obtained in a young woman shows the lungs. **(b)** Photograph shows the patient with appropriate shielding of the lower abdomen and pelvis. Proper shielding is necessary to both reassure the patient and minimize radiation exposure. Documenting such maneuvers may become necessary from a medicolegal standpoint.

**Figure 4.**
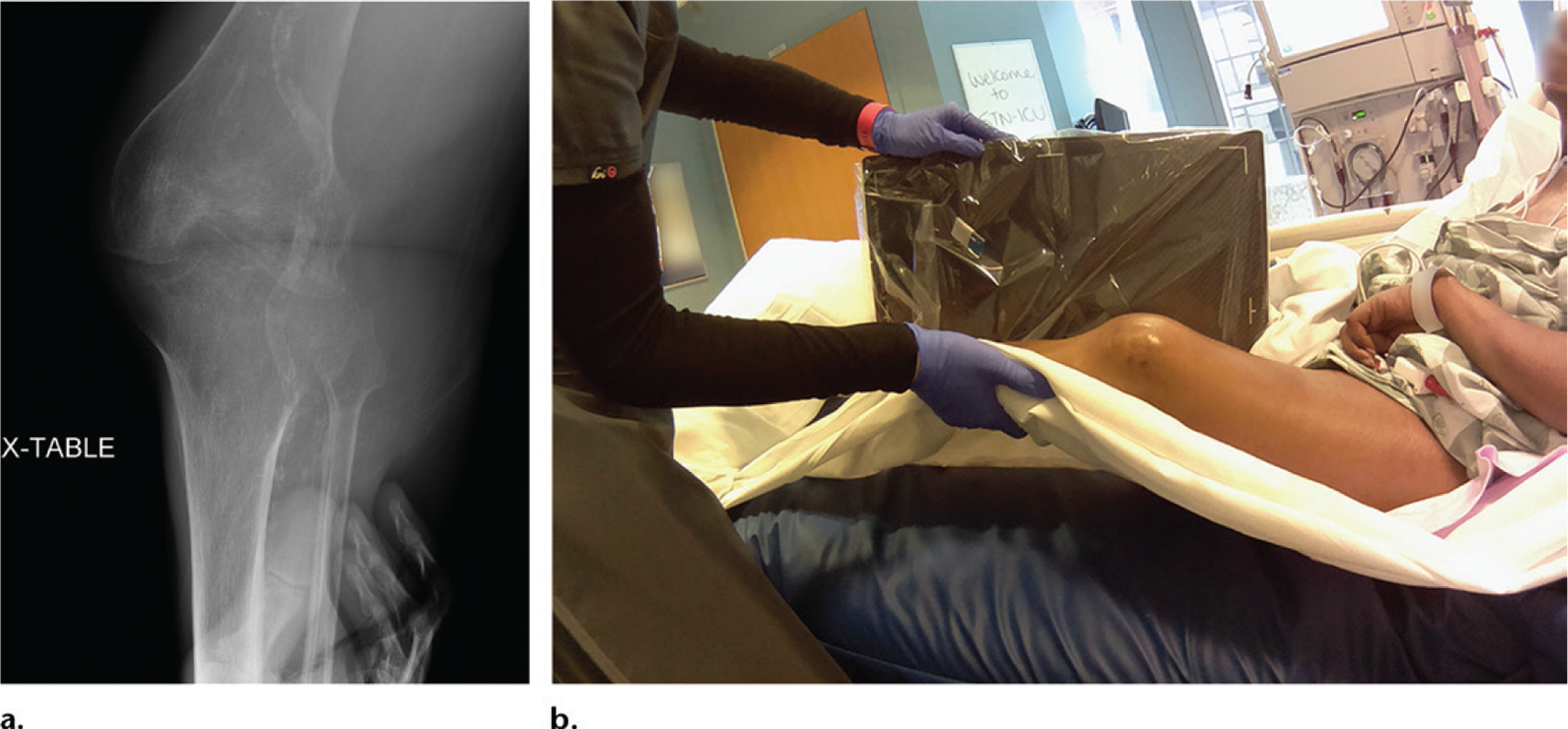
**(a)** Cross-table lateral radiograph of the knee obtained in a suboptimal patient position. Laterality information was not provided on the radiograph. **(b)** Photograph shows the physical challenges the technologist faced in properly obtaining this view in this patient, who could not cooperate. The photograph documents the medicolegal explanation for the suboptimal examination, while making it clear that the left knee was being imaged.

Point-of-care photographs provide information regarding patient position and any precautions that the radiologic technologist may have taken during acquisition of the images. For example, placement of lead shielding by technologists over the lower abdomen and pelvis of younger patients may become necessary from a medicolegal standpoint in the future (Fig 3). Such documentation can be useful for quality control monitoring and improvement. In several instances, technologists who experienced this camera technology were gratified that the difficulties in acquiring certain views in certain patients were evident on the photographs (Fig 4), and radiologists were considering information on these photographs when providing quality control comments through the electronic feedback tools.

**Figure 5.**
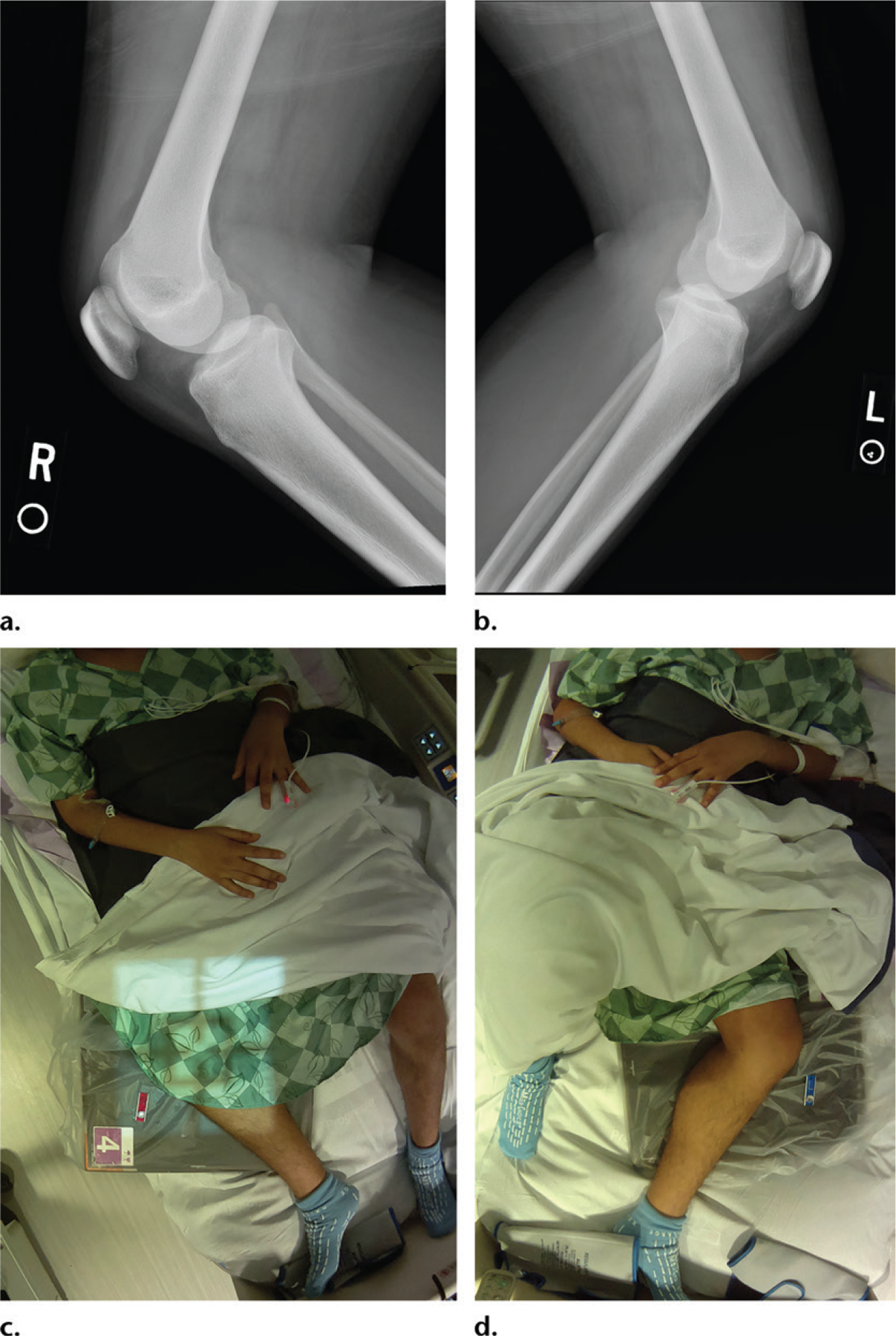
**(a, b)** Lateral radiographs show the right **(a)** and left **(b)** knees in a man with knee pain. **(c, d)** Photographs show the patient at imaging, demonstrating that the right **(c)** and left **(d)** knees were imaged. This confirms that the correct side was imaged in each case.

In addition, laterality issues are largely solved by the technology because the wide-angle lens used to obtain the photographs includes a substantial portion of the patient. In several cases, particularly when bilateral extremities were imaged, radiologists were confident about the side that they were interpreting when the simultaneously obtained photograph was also available (Fig 5). The order of acquisition of the radiographs is mirrored by the order of acquisition of the photographs to avoid confusion.

These examples show that obtaining point-of-care patient photographs can be useful for documenting patient conditions and for quality assurance purposes.

## Assisting with Interpretation

**Figure 6.**
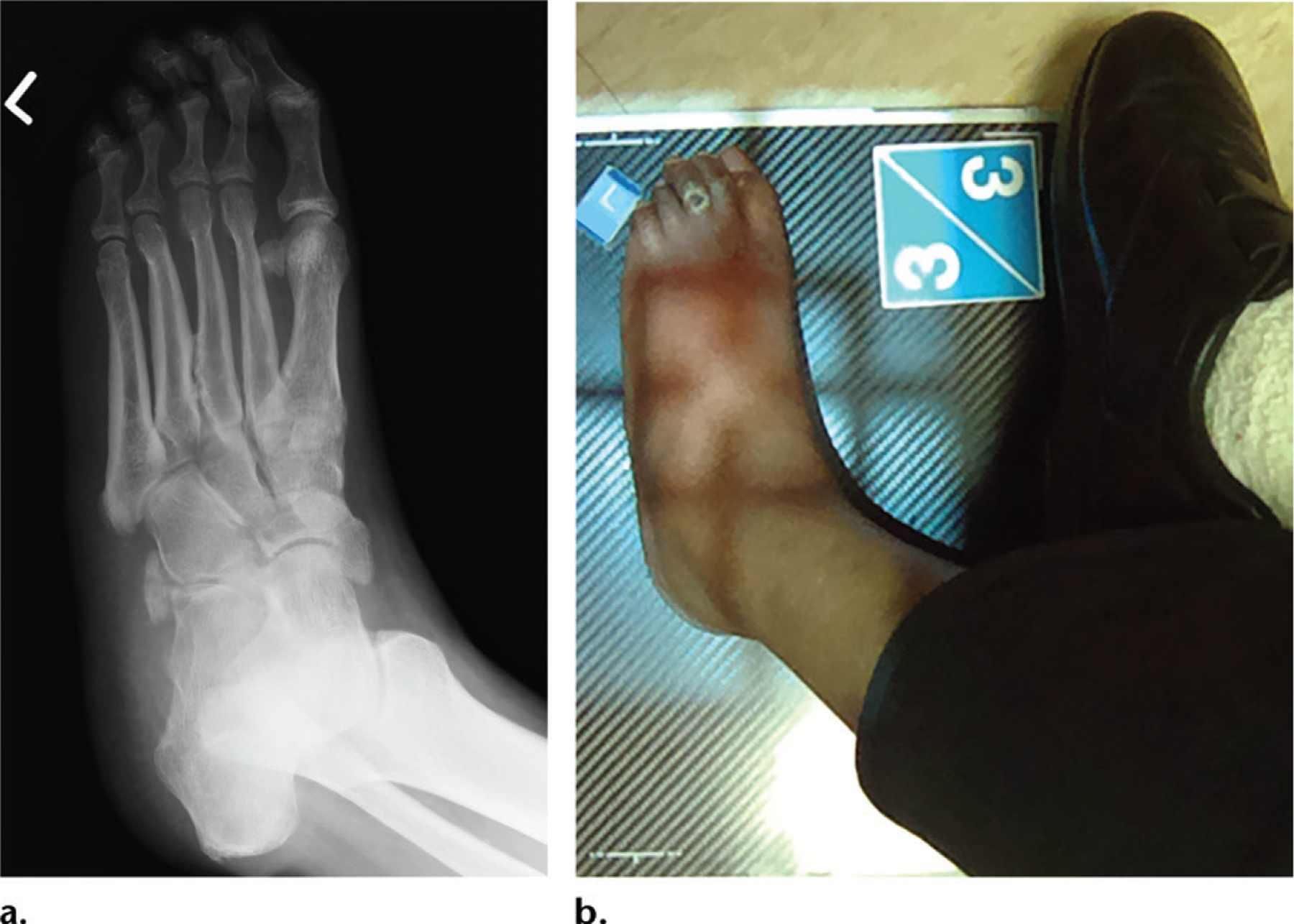
**(a)** Oblique radiograph shows the left foot in a 46-year-old man who underwent imaging for possible osteomyelitis related to a diabetic foot ulcer. The interpretation can be substantially accelerated if there is an indication of the location of the ulcer. **(b)** Coned-down photograph shows the ulcer on the third toe of the left foot, which helps focus the radiographic evaluation.

**Figure 7.**
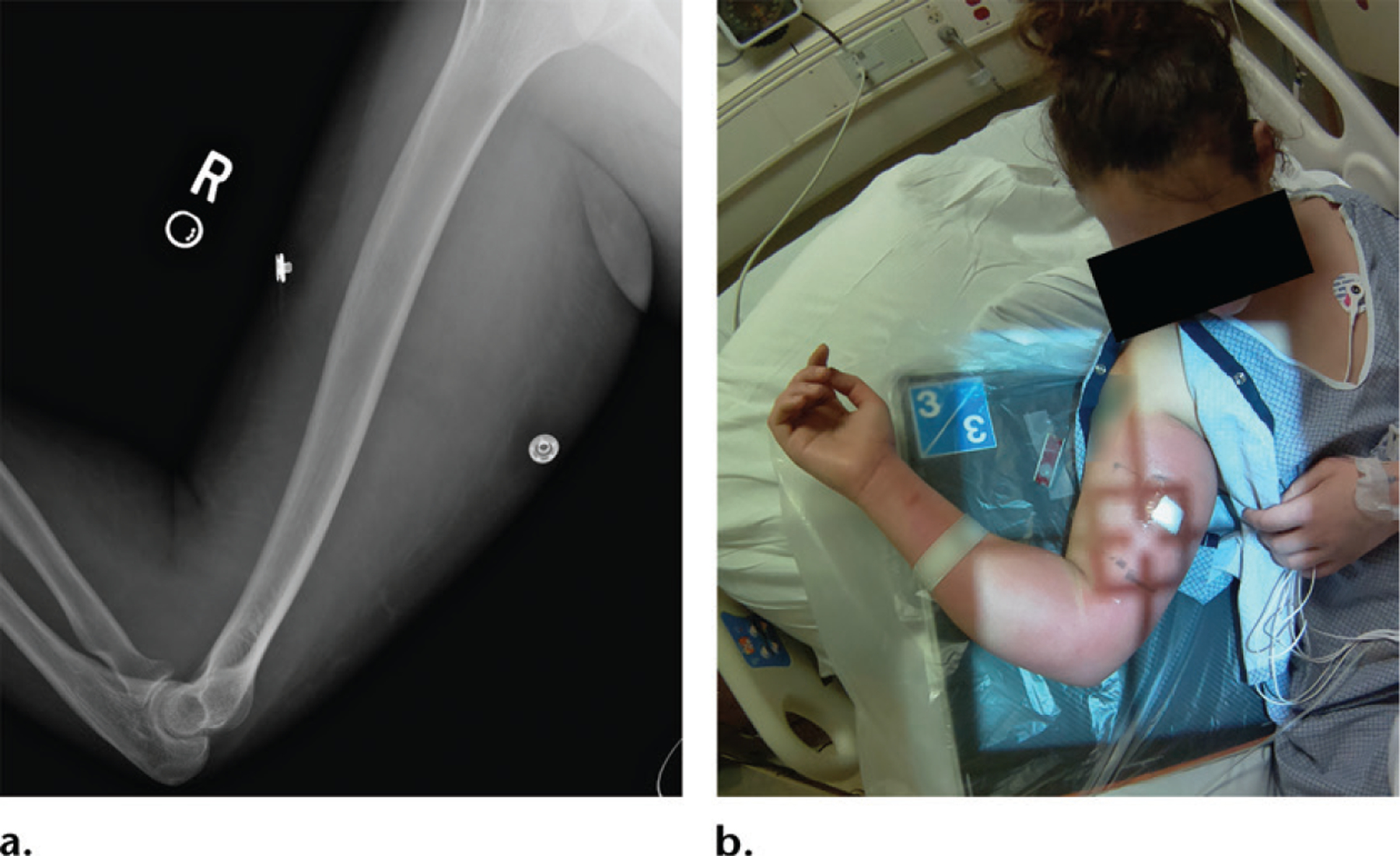
Radiograph **(a)** and patient photograph **(b)** show the right humerus in a woman with pain. Soft-tissue swelling was not noted at the initial evaluation of the radiograph but was noted on the final report after the radiologist integrated information obtained from the photograph. The soft-tissue swelling and erythema, findings consistent with cellulitis, were not noted in the provided clinical history. However, these findings are obvious on the photograph. This information could prompt the radiologist to evaluate for osteomyelitis rather than a fracture.

In musculoskeletal radiology, a common reason for obtaining lower extremity radiographs is to evaluate for osteomyelitis in the setting of diabetic foot ulcers. Radiologists often lament the lack of clinical information regarding the location of the ulcers when interpreting these radiographs. When the location of the ulcer is known, the interpretation can be hastened, as acute osteomyelitis if present is unlikely to manifest far from the ulcer (Fig 6). Likewise, soft-tissue swelling and erythema visualized on point-of-care patient photographs can provide clinical information that may not have been included with the requisition (Fig 7).

**Figure 8.**
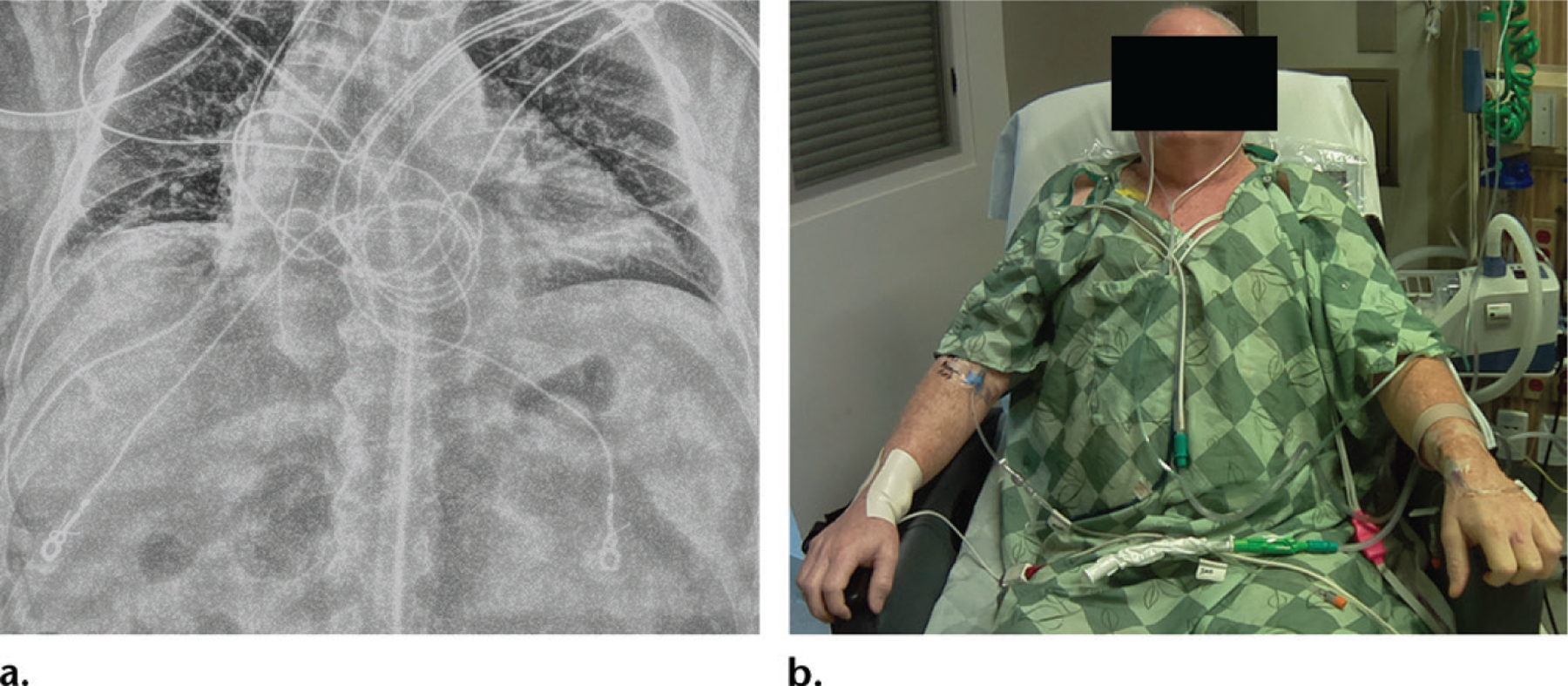
**(a)** Frontal abdominal radiograph obtained to exclude a diagnosis of pneumoperitoneum shows no free air. However, it was unclear from the radiograph whether the image was obtained with the patient in a supine or upright position, as the radiopaque marker was missing. **(b)** Photograph shows the patient in a near-erect position, adding confidence to the impression that there is no free air on the radiograph.

Abdominal radiographs are often obtained to rule out pneumoperitoneum. In many instances, if the gravity-dependent marker is not included in the radiograph, then it may be difficult to rule out free air confidently, as patient position (supine vs upright) cannot be ascertained. Even if the marker is placed on the radiographic detector, the patient’s position may not correlate with that of the marker. The photograph can show whether the patient is supine or upright and add confidence to the interpretation (Fig 8).

**Figure 9.**
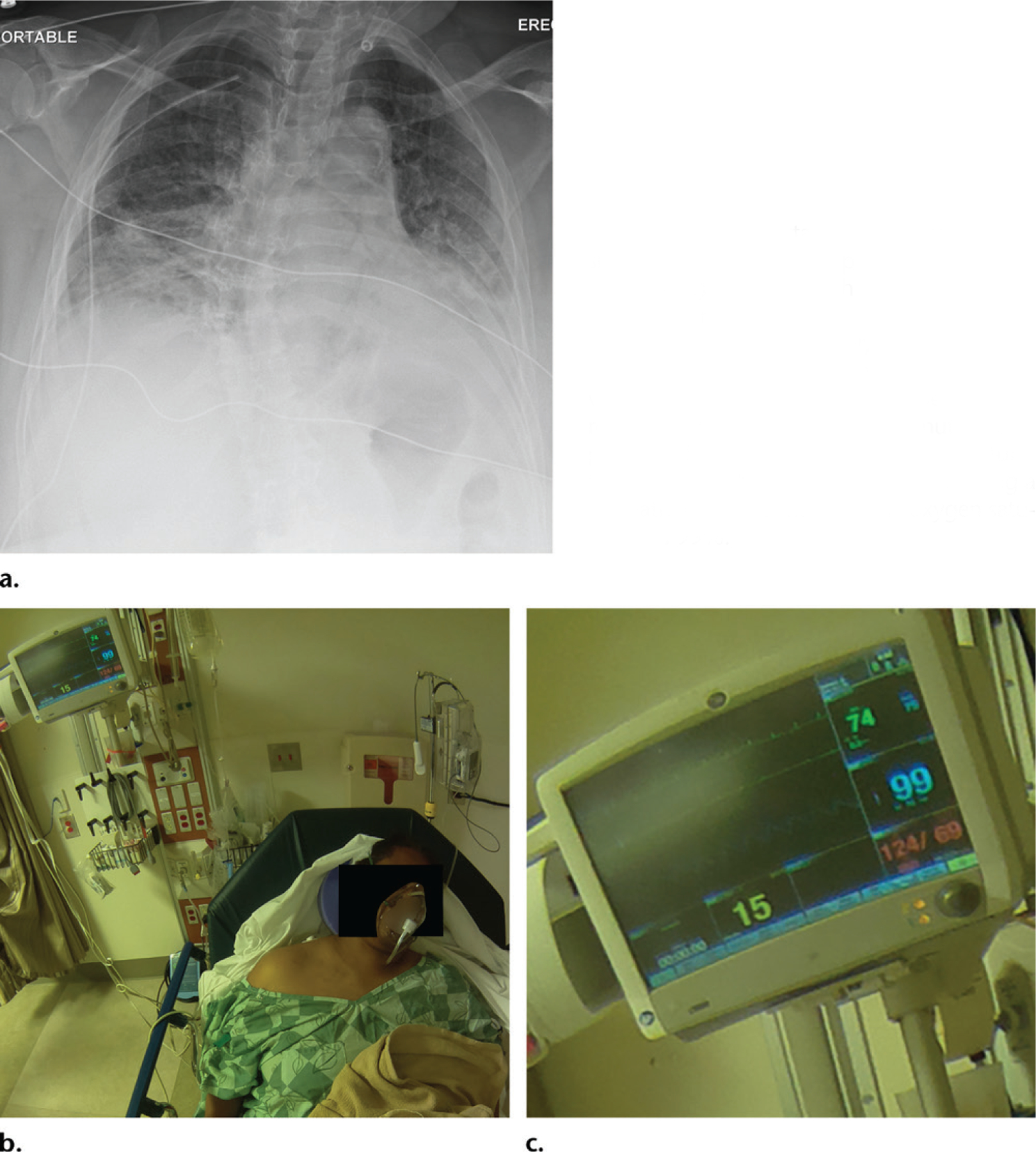
**(a)** Portable chest radiograph shows bibasilar airspace opacities. **(b, c)** Photograph **(b)** obtained with a wide-angle lens shows that most of the room was imaged, including the patient monitor, which has been magnified **(c)** to show the patient’s stable vital signs (heart rate, 74 beats per minute; respiratory rate, 15 breaths per minute; blood pressure, 124/69 mm Hg; and oxygen saturation, 99%). Note that the patient was using a rebreather mask to maintain the oxygen saturation of 99%.

**Figure 10.**
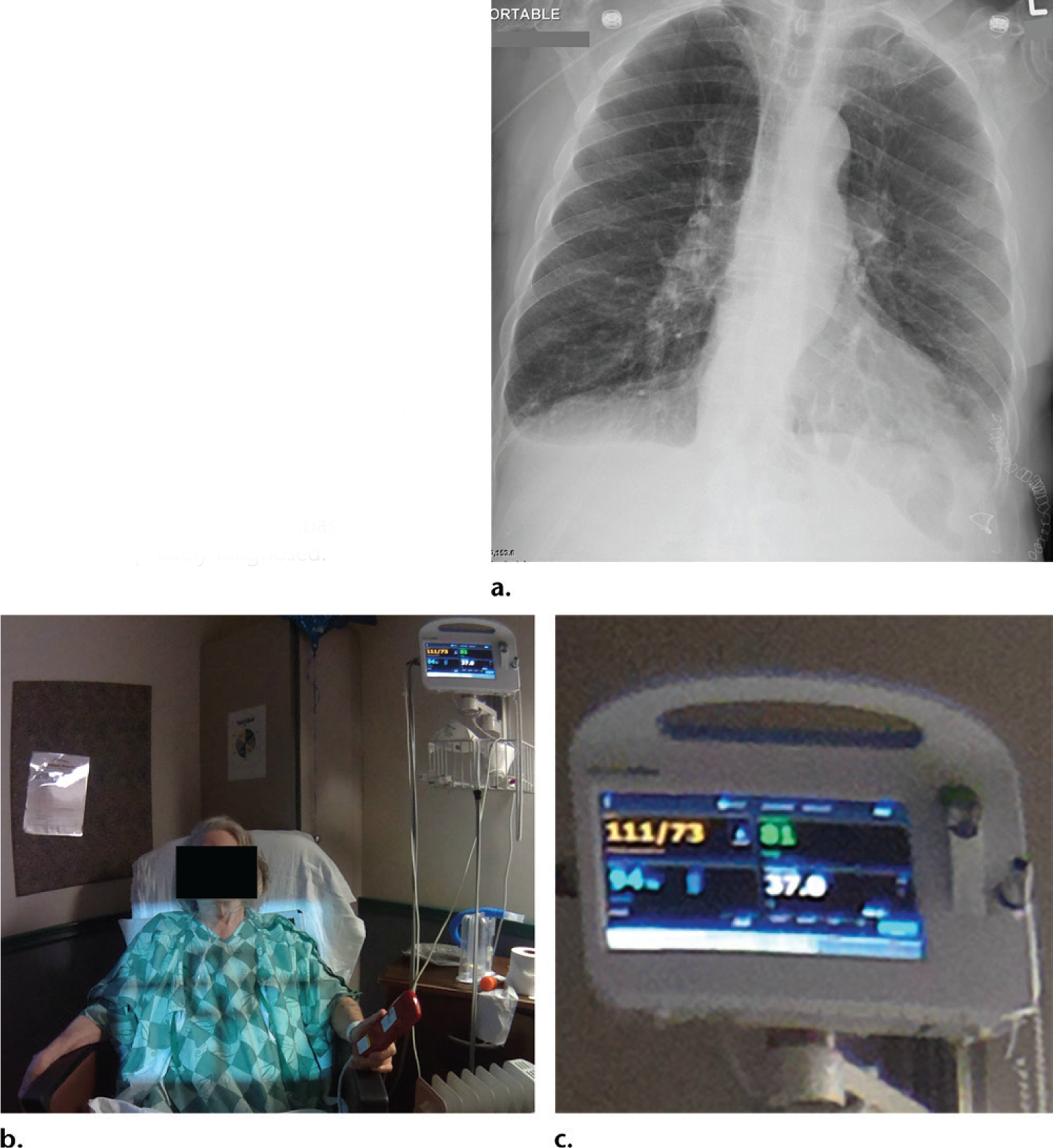
**(a)** Radiograph obtained to rule out pneumonia shows small pleural effusions and minimal bibasilar airspace opacities. **(b, c)** Corresponding photograph **(b)** with a magnified view of the patient’s monitor **(c)** show the patient’s vital signs (heart rate, 81 beats per minute; blood pressure, 111/73 mm Hg; oxygen saturation, 94%; and temperature, 37.0° C). The patient’s normal temperature indicated that a diagnosis of pneumonia was not correct, and bibasilar atelectasis was appropriately diagnosed.

Finally, as wide-angle lenses used in the camera technology also capture a substantial portion of the hospital room, patient monitors in the room may also be visible. These monitors can include information such as the patient’s heart rate, respiratory rate, oxygen saturation, blood pressure (Fig 9), and temperature (Fig 10) at the time of imaging. For example, awareness of the patient’s temperature can be useful when interpreting a lung opacity. Knowledge that the patient is febrile can increase the diagnostic certainty that the finding is related to an infectious cause. While it can be argued that such information should be available in the patient’s electronic medical record, such information may not be easy or efficient to obtain in many environments. The photograph allows the information to be embedded in the images in the PACS and can be presented along with the radiographs at the time of interpretation, facilitating integration of such data.

One note of caution is that the data on the patient monitors may not be current and could potentially be from another patient. If this type of information is to be widely used, the monitors may need to be redesigned to display the time of the acquisition of each instance of data (eg, heart rate, respiratory rate) and the patient’s name.

On the basis of the information shown in these examples, point-of-care patient photographs can provide image-related clinical context that can increase confidence in radiographic diagnosis.

## Preventing Unnecessary Calls: Performing One’s Own Clinical Correlation

A significant part of interpreting portable chest and abdominal radiographs involves the assessment of support hardware, such as central venous catheters, endotracheal tubes, and enteric tubes, among others.

**Figure 11.**
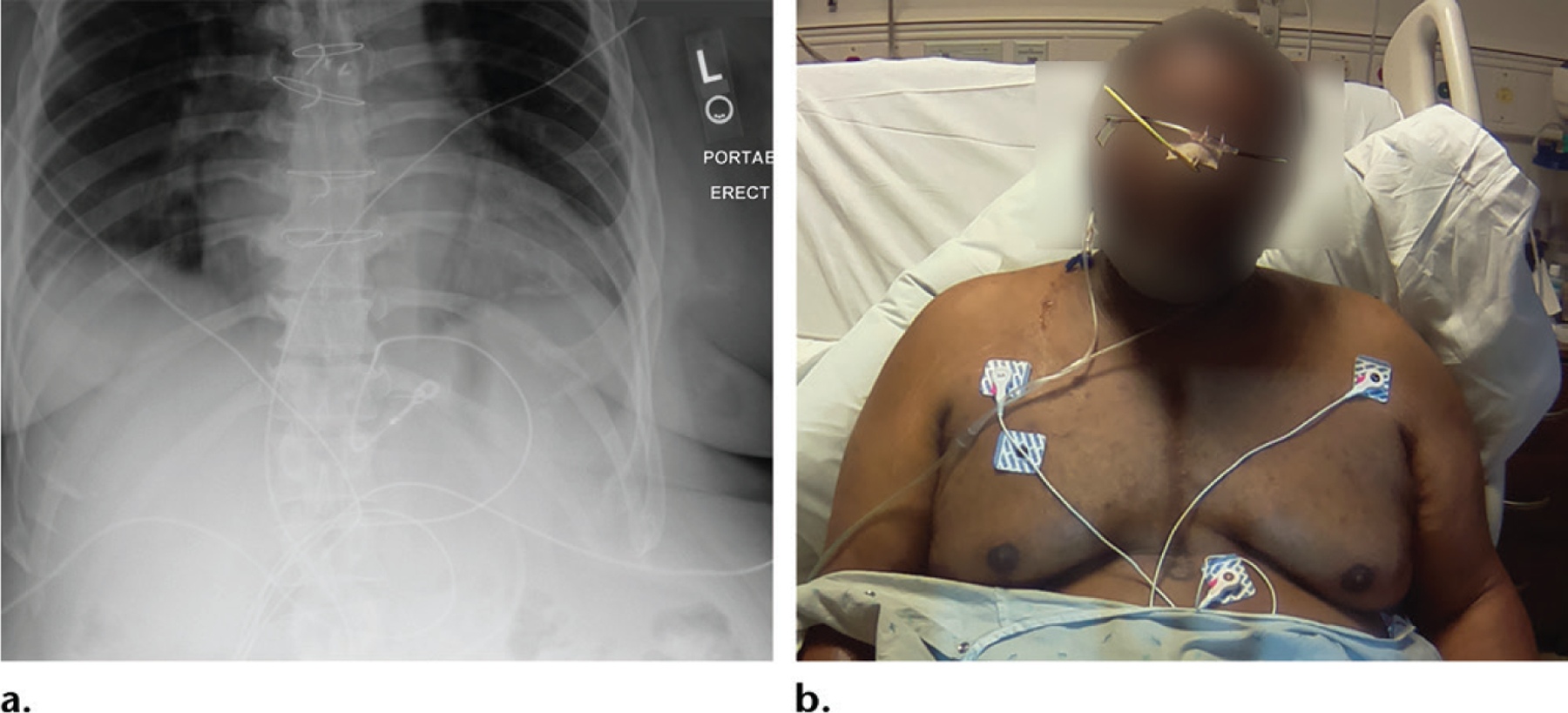
Thoracoabdominal radiograph obtained to evaluate for feeding tube placement. **(a)** Radiograph does not show a feeding tube, a finding that would have prompted the radiologist to call the floor nurse to ascertain if a tube was in place. **(b)** Photograph shows the patient with the feeding tube, which has possibly coiled in the upper chest or neck. This requires an urgent and confident call from the radiologist to reposition the tube.

**Figure 12.**
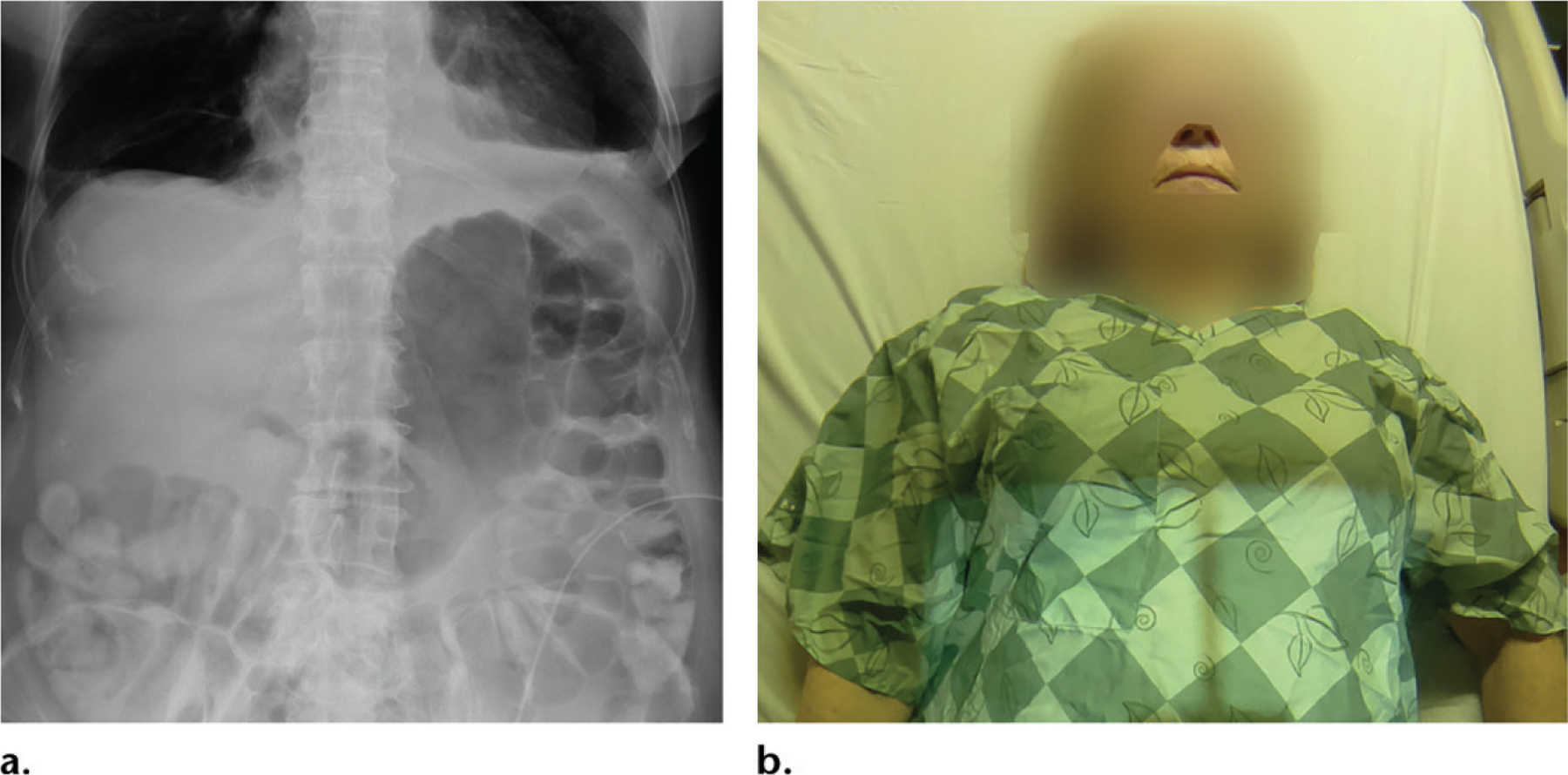
**(a)** Thoracoabdominal radiograph obtained to evaluate nasogastric tube position does not show an enteric tube in the lower chest or upper abdomen. The tube could potentially be coiled in the upper mediastinum or neck. **(b)** Photograph shows the patient with no tube entering the nares or the mouth. A call to the patient’s nurse or clinical service can be avoided with the review of point-of-care patient photographs.

Quite often, thoracoabdominal radiographs, which image the lower thorax and upper abdomen, are obtained to ascertain the placement of enteric tubes (eg, feeding or gastric tubes). A substantial portion of these radiographs are obtained because of prior standing orders, ordered at a time when the patient may have had an enteric tube, and the patient may not have such hardware at the time of imaging. Thus, if an enteric tube is not visualized on the thoracoabdominal radiograph, the interpreting radiologist may be unsure if a tube is present or not. If a tube is indeed present, as seen in Figure 11b, then lack of visualization may imply that the tube is either in the upper chest or coiled in the neck. Both conditions would require an urgent call to the patient’s clinical service to have the tube repositioned. However, if the tube is not present, then a call to the patient’s service would be a wasted effort (Fig 12).

**Figure 13.**
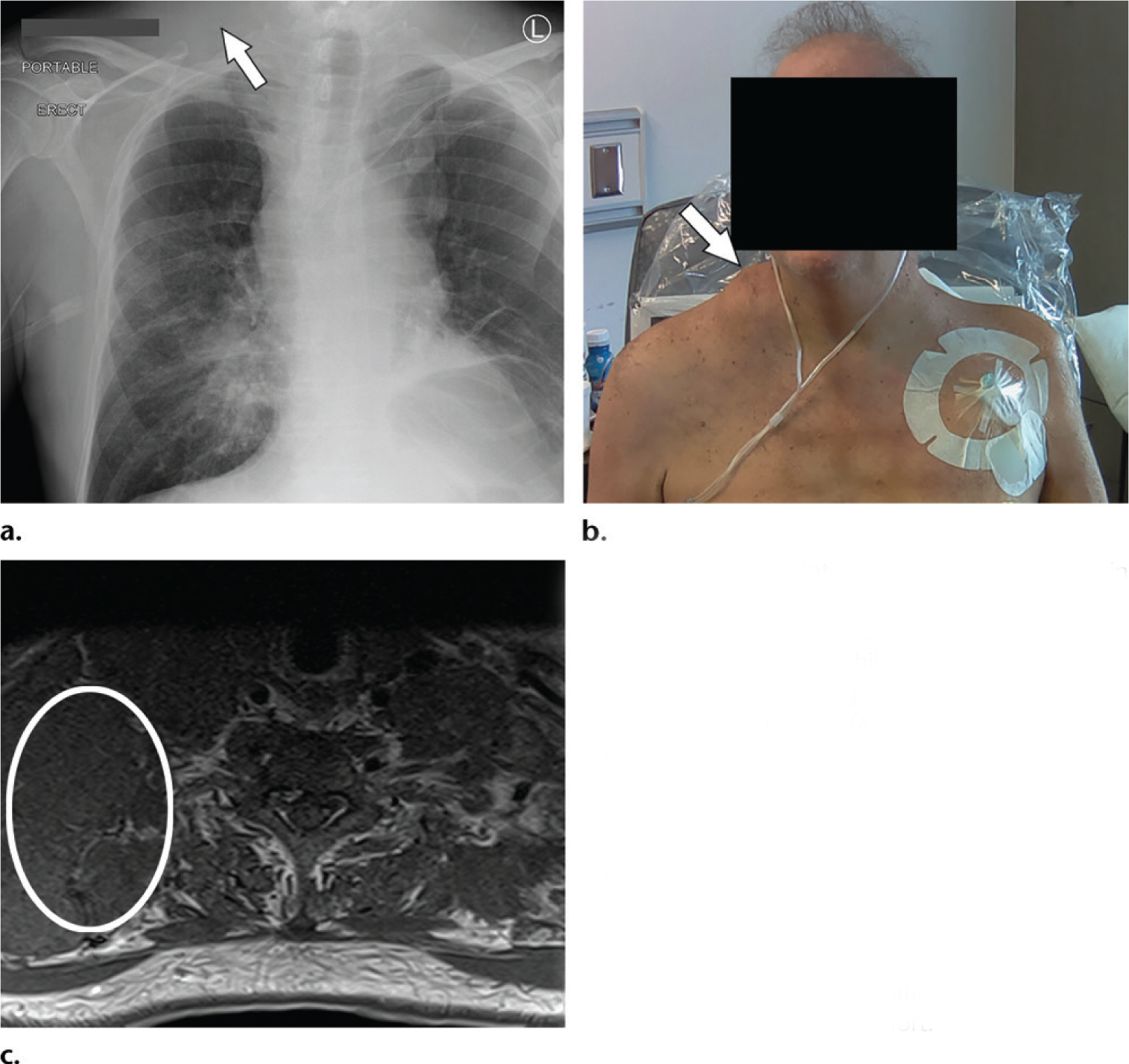
Metastatic spread of primary cancer in a man who underwent portable chest radiography for evaluation of chest port placement. **(a)** Frontal radiograph shows right hilar fullness and elevation of the left hemidiaphragm. **(b)** Photograph shows asymmetric soft tissue (arrow in **a** and **b**) in the right suprascapular region, which is not as obvious on the radiograph. **(c)** Axial MR image shows a right neck mass (circle). Although information about the right neck mass was available on the MR image obtained prior to the radiograph, the radiologist interpreting the portable radiograph would not be likely to open the MRI study. The findings on the photograph could prompt the radiologist to report a possible metastatic nodal mass in the right neck without much effort.

As with the clinical context provided for musculoskeletal examinations, soft-tissue findings may also be useful in the interpretation of chest and abdominal radiographs. In Figure 13b, the point-of-care patient photograph shows a focal mass in the right neck, which is not obvious on the radiograph. This photograph could alert the interpreter that the malignancy has possibly spread to the right neck and is thus metastatic.

## New Way to Physically Assess Patients

**Figure 14.**
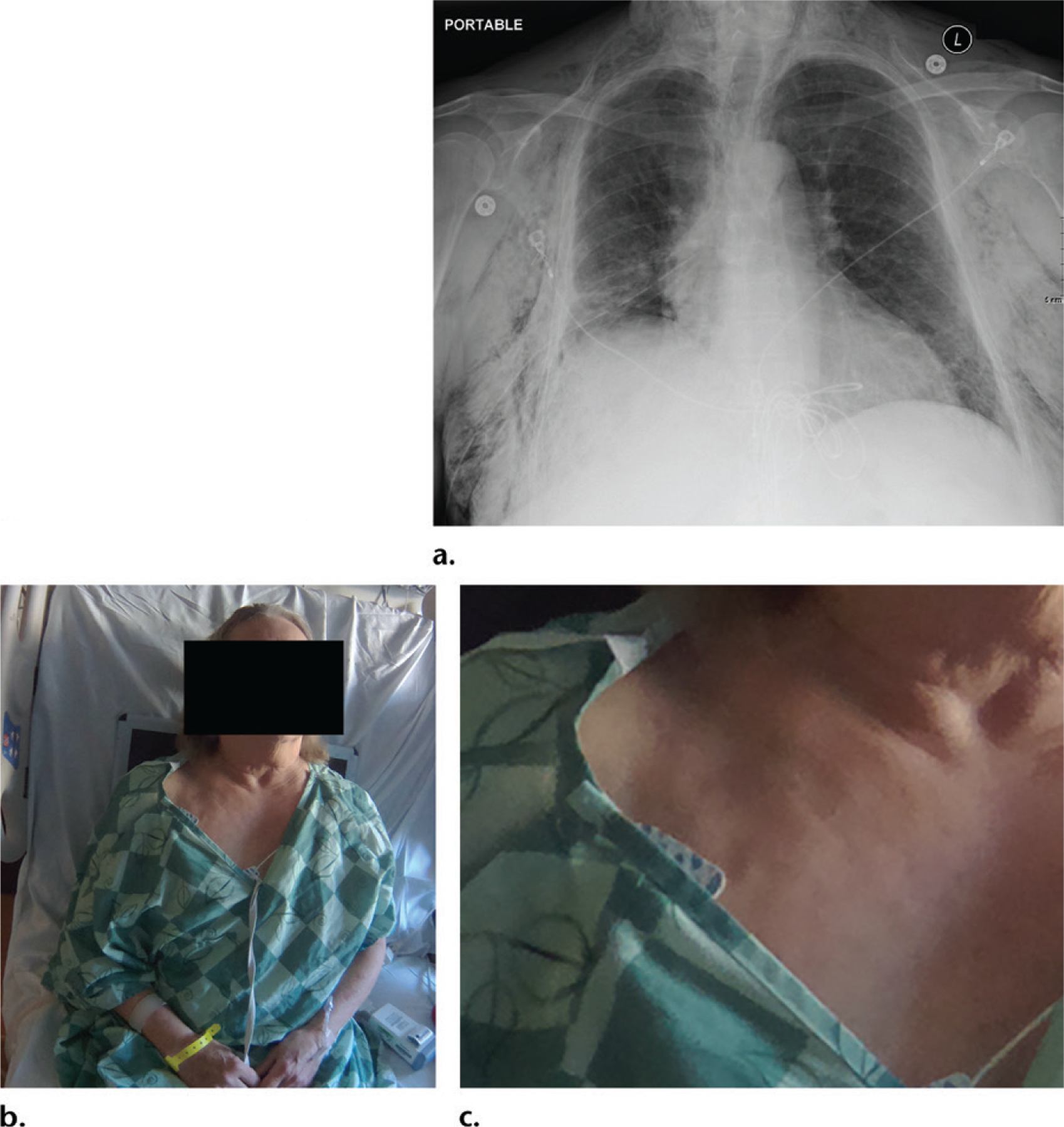
**(a)** Radiograph shows subcutaneous emphysema from an air leak following right lower lobectomy for malignancy. **(b, c)** Photograph **(b)** and the coned-down magnified view **(c)** of the upper chest shows the uneven skin surface, a finding suggestive of subcutaneous emphysema.

An unexpected finding in our initial experience was that the photographs could potentially provide new ways to physically assess patients. In one striking example (Fig 14), a patient with substantial subcutaneous emphysema following thoracotomy had textural changes to the skin surface that were visible on the accompanying patient photograph. While crepitus related to subcutaneous emphysema is a well-known finding on palpation, subcutaneous emphysema could potentially be noted as a finding at physical examination. The technology may lead to unprecedented avenues for future clinical assessment, as it now allows for correlation of the patient’s external appearance (photographs) with the internal findings (radiographs).

With the development of machine learning techniques, correlating radiographic findings with photographic findings could lead to incorporation of physical findings at interpretation, which were hitherto ignored because the importance of these findings was not known. It is possible that, in the future, photographs could be used in lieu of radiographs, which require the use of ionizing radiation, for certain restricted indications.

## Photography in Medicine and Other Approaches to Photography in Radiology

The use of photography in other areas of medicine has been well documented. Nearly 30 years ago, a method of obtaining total-body photographs to document dysplastic nevi was described (12). It was noted that a comparison between the baseline photographs and subsequent clinical findings can permit detection of thin malignant melanomas in a curable stage. A prototype 16-camera imaging system was used to image 11 subjects who had a total of 110 lesions, and an observer study with dermatologists showed 100% sensitivity for the detection of easy lesions and 98% sensitivity for the detection of difficult lesions, where the difficulty was determined mainly on the basis of lesion size and the subjective assessment of visibility (13). Indeed, total-body photography is now reimbursable by insurance providers when employed to evaluate patients with a history or a close family history of atypical nevi, dysplastic nevi, melanoma, or nonmelanoma skin cancers. Digital photography is also well established as a method to track wound care, and standardized wound photography protocols for accurate and objective recording have been proposed (14–16).

Facial photograph phenotyping has also been used to study various disease processes and syndromes. For example, Astley and Clarren (17) noted that the phenotypic case definition derived from photographs accurately distinguished between those patients with and those without fetal alcohol syndrome. Reither et al (18) discussed extant literature describing that facial and neck characteristics are related to body mass and central adiposity. Motivated by this observation, the authors developed a substitute measure of body mass on the basis of facial features.

More closely related to our application, facial photographs have been suggested as an identification tool for computerized–provider order entry (CPOE) systems. Hyman et al (19) showed that including patient photographs within a CPOE verification process in a pediatric hospital was effective in reducing the risk that orders would be placed erroneously in the incorrect patient’s chart. Through a simulated study, Taieb-Maimon et al (20) demonstrated that the addition of patient photographs and a highlighted menu selection could substantially reduce errors in CPOE systems and other computerized applications.

Within radiology, at least one PACS vendor and vendors of smart phones or tablets have provided tools to capture photographs or visible light images and transfer and store them in a PACS or an electronic medical record. Such tools allow for the use of mobile devices to capture images that can be transferred to the PACS. Of note, such systems require human intervention and do not capture photographs automatically. Similarly, the data (ie, the patient photographs) from our previously published articles on observer studies were obtained manually using a mobile phone at the time of portable radiography (7–9,11). A second disadvantage with this prior approach and with such mobile-device photograph-capture applications is that the photographs are not acquired simultaneously with the radiograph acquisition. Thus, a true correlation between the photographs and radiographs is not guaranteed.

## Conclusion

We clinically implemented a system for automatically and seamlessly obtaining point-of-care patient photographs simultaneously with portable radiographs. Such photographs can provide various benefits, including increasing the detection rate of wrong-patient errors and providing image-related clinical context. The latter can lead to improved and potentially faster interpretation. In addition, such photographs allow radiologists to correlate the external appearance of patients with the radiographic findings. We speculate that such correlations could be used in the future with machine learning techniques to potentially develop new ways of externally assessing patients without the use of ionizing radiation.

## SA-CME LEARNING OBJECTIVES

After completing this journal-based SA-CME activity, participants will be able to:

- Recognize that wrong-patient errors continue to occur in radiology, despite the use of the Joint Commission–mandated dual-identifier technique.
- Explain how point-of-care patient photographs obtained at the time of radiography can increase detection of wrong-patient errors.
- Discuss how patient photographs can provide clinical context and increase confidence in radiographic interpretation.

*See* rsna.org/learning-center-rg.

## TEACHING POINTS

- Despite use of the Joint Commission–mandated dual-identifier technique for positive patient identification, wrong-patient errors continue to occur in radiology.
- Patient photographs obtained at point-of-care portable radiography can help identify wrong-patient errors.
- Point-of-care photographs provide information regarding patient position and any precautions that the radiologic technologist may have taken during acquisition of the images.
- Obtaining point-of-care patient photographs can be useful for documenting patient conditions and for quality assurance purposes.
- Point-of-care patient photographs can provide image-related clinical context that can increase confidence in radiographic diagnosis.

## Abbreviations:

DICOM: Digital Imaging and Communications in Medicine
IS: integration server
PACS: picture archiving and communication system
